# A short review of fecal indicator bacteria in tropical aquatic ecosystems: knowledge gaps and future directions

**DOI:** 10.3389/fmicb.2015.00308

**Published:** 2015-04-17

**Authors:** Emma Rochelle-Newall, Thi Mai Huong Nguyen, Thi Phuong Quynh Le, Oloth Sengtaheuanghoung, Olivier Ribolzi

**Affiliations:** ^1^iEES-Paris, UMR 7618 (IRD-UPMC-CNRS-INRA-Université Paris-Est, Université Paris 7), Centre IRDBondy, France; ^2^Institute of Natural Products Chemistry, Vietnam Academy of Science and TechnologyHanoi, Vietnam; ^3^Agriculture Land Research Center, National Agriculture and Forestry Research InstituteVientiane, Laos; ^4^Institut de Recherche pour le Développement, Géosciences Environnement Toulouse, UMR 5563, Université Paul SabatierToulouse, France

**Keywords:** FIB, *E. coli*, biotic and abiotic factors, die-off rates, developing countries, millennium development goals, hydrological modeling

## Abstract

Given the high numbers of deaths and the debilitating nature of diseases caused by the use of unclean water it is imperative that we have an understanding of the factors that control the dispersion of water borne pathogens and their respective indicators. This is all the more important in developing countries where significant proportions of the population often have little or no access to clean drinking water supplies. Moreover, and notwithstanding the importance of these bacteria in terms of public health, at present little work exists on the persistence, transfer and proliferation of these pathogens and their respective indicator organisms, e.g., fecal indicator bacteria (FIB) such as *Escherichia coli* and fecal coliforms in humid tropical systems, such as are found in South East Asia or in the tropical regions of Africa. Both FIB and the waterborne pathogens they are supposed to indicate are particularly susceptible to shifts in water flow and quality and the predicted increases in rainfall and floods due to climate change will only exacerbate the problems of contamination. This will be furthermore compounded by the increasing urbanization and agricultural intensification that developing regions are experiencing. Therefore, recognizing and understanding the link between human activities, natural process and microbial functioning and their ultimate impacts on human health are prerequisites for reducing the risks to the exposed populations. Most of the existing work in tropical systems has been based on the application of temperate indicator organisms, models and mechanisms regardless of their applicability or appropriateness for tropical environments. Here, we present a short review on the factors that control FIB dynamics in temperate systems and discuss their applicability to tropical environments. We then highlight some of the knowledge gaps in order to stimulate future research in this field in the tropics.

## Introduction

**FIGURE 1:**
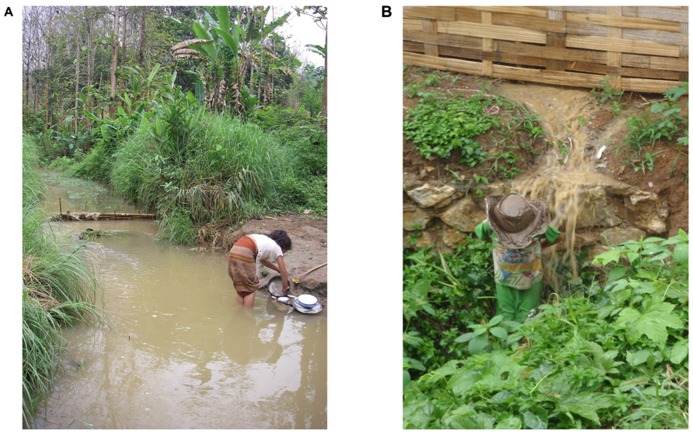
**In developing countries the lack of adequate infrastructures means that contaminated water is used for domestic activities (A).** During the rainy season when river and stream flow is high and temperatures are elevated children often play in water contaminated with latrine overflows **(B)**.

In many countries, poor water quality continues to pose a major threat to human health and access to clean drinking water and adequate sanitation continues to be a major brake on development. It is estimated that on a global scale, diarrheal diseases are responsible for deaths of 1.8 million people annually, most of whom are children from developing countries (WHO, 2012). An estimated 88% of that burden is directly attributable to unsafe water supply, sanitation and hygiene. Indeed, in most developing countries access to clean drinking water and adequate sanitation remains a problem despite increases in recent years. The economic situation and lack of effective infrastructure means that a large proportion of the population relies on untreated surface and groundwater that can be highly contaminated. Moreover, river water subject to wastewater contamination is often used for washing of clothes and food utensils and for bathing and even cooking (Bain et al., 2014b; **Figure 1**). This is true for urban and peri-urban areas where population densities are high (Ashbolt, 2004; Kimani-Murage and Ngindu, 2007; Opisa et al., 2012; Bain et al., 2014b) as well as in rural areas where water supplies are often informal and therefore unregulated. Recent work has found that rural drinking water was more contaminated than that of urban water supplies in some parts of Africa and Asia (Bain et al., 2014a,b; Christenson et al., 2014). These authors found that over half the drinking water sources tested in Africa were contaminated as compared to 35% in Asia and that on average, over 40% of rural drinking water sources are contaminated as compared to only 12% in urban areas. Access to clean water is therefore a problem faced by both urban and rural populations in developing countries.

Developing countries are faced by a double problem. Often no adequate structures exist for the long term monitoring of water borne pathogens in the environment due to economic constraints and secondly, very little knowledge exists on the distribution of these microbes in tropical environments. Yet, considering the known risks associated with the consumption of sewage contaminated water, it is critical that the factors that control the persistence and dissemination of these microbial pathogens in developing countries are identified. Moreover, by increasing the knowledge base on the dynamics of the water borne pathogens in tropical ecosystems we will be able to reduce the risks associated with the use of untreated water. However, at present, most existing work in the tropics tends to focus on drinking water supplies (bore holes, well, etc.) with the general environment being ignored (Opisa et al., 2012; Bain et al., 2014a) and this despite the importance of these informal water sources for domestic use.

Tropical ecosystems are generally characterized by higher and more stable temperatures, higher light intensities, and lower variability in day length as well as, in the case of humid tropical regions, high relative humidity when compared to temperate ecosystems. All of which can be strong drivers of ecological diversity and activity. This is all the more important for microbial (pathogen and non-pathogen) species for which environmental stability or instability can be a strong factor in determining diversity (De Wit and Bouvier, 2006). Strong latitudinal gradients in diversity have been observed for natural aquatic bacterial communities (e.g., Pommier et al., 2007); Guernier et al. (2004) also reported this for pathogenic bacteria and viruses. However, this does not appear to be the case for soil bacteria that are more influenced by the edaphic factors rather than temperature (Fierer and Jackson, 2006). Nevertheless, the environmental differences between temperate and tropical ecosystems may well mean that water borne microbial pathogens will have different behaviors in tropical, humid systems and therefore the application of temperate models in the tropics may not be appropriate.

The pathogens (bacteria, viruses, and protozoa) responsible for these water borne diseases are also susceptible to shifts in hydrology and water quality (Vidon et al., 2008b; Cho et al., 2010a; Ratajczak et al., 2010; Chu et al., 2011; Liang et al., 2013). This is particularly important for developing countries as the anticipated increases in rainfall and floods due to climate change will also lead to the washing out of fecal matter from latrines on land into drinking water supplies through contamination of bore holes and of reservoirs. This will be further exacerbated by the rapid shifts in land use that many tropical regions are experiencing. Moreover, increasing urbanization and the need for clean water and adequate sanitation are only going to accelerate. Therefore recognizing and understanding the link between human activities, natural process and hydrological and biogeochemical functioning and their ultimate impacts on human health are prerequisites for efficient water resources management.

In order to detect these waterborne pathogens at limited cost, fecal indicator bacteria (FIB) are used as a proxy for pathogenic bacteria. The term FIB describes the range of bacteria that inhabit the gastrointestinal tract of homeothermic animals and includes *Escherichia coli* and the fecal coliforms, *Enterococcus* spp., all of which are permanently excreted in fecal material. Here, we present a short review on FIB in tropical ecosystems. Our goal is not to repeat what has already been published on temperate systems (e.g., Ferguson and Signoretto, 2011; Pachepsky et al., 2011) but to focus on the specificities of tropical ecosystems and to highlight some of the present knowledge gaps and to suggest some potential new directions for research in this area.

## FIB: Application to the Tropics

Fecal indicator bacteria are used as a proxy for detecting the presence of pathogenic bacteria in environmental samples such as soil and water and therefore should behave in the same way as the pathogens there are supposed to be a proxy for. Ideally, indicator bacteria should be present in the intestinal tract of the same animal as the pathogens; should be present only in contaminated samples and not in uncontaminated ones; should have similar survival patterns as pathogens outside the host; should not be able to grow and proliferate in the environment; should be easily detectable; be of low risk to the person conducting the analyses and, ideally, should be relatively cheap to use (Ishii and Sadowsky, 2008; Ferguson and Signoretto, 2011).

**FIGURE 2:**
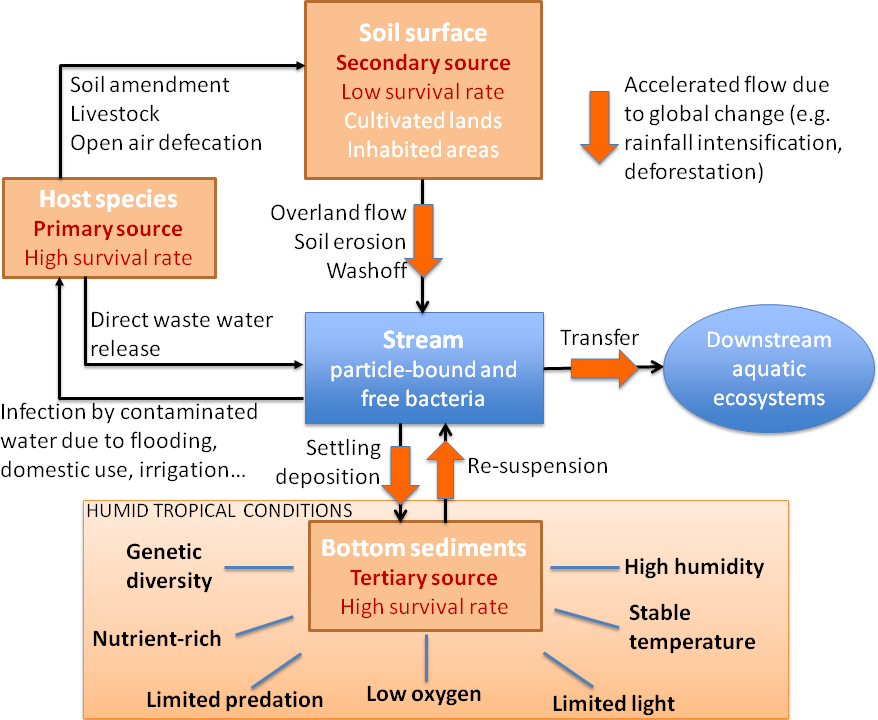
Conceptual diagram of the primary, secondary, and tertiary sources of FIB in humid tropical environments. Humans and livestock are the primary sources of FIB and are responsible for the direct contamination of soils and water. The secondary reservoir is comprised of cultivated land and inhabited rural areas that have been contaminated by the primary sources. The tertiary source is comprised of stream bed sediments and hyporheic zones that offer conditions that are more similar to the host intestinal tract: low or no light due to the density of riparian vegetation, lower oxygen conditions, reduced predation by protozoans, and higher organic matter and nutrient concentrations.

The criteria used to select FIB are based on the application of the above points as determined for temperate systems. The organisms chosen (fecal coliforms or *E. coli*, for example) are then subsequently applied to tropical systems without taking into account the potential specificities of the tropics (such as higher temperature and humidity, differences in nutrient and organic matter availability and higher solar irradiation levels). All of which may affect the persistence and hence, utility of the selected bacteria as an FIB. This is further compounded by the fact that it is not entirely clear that indicator bacteria used in temperate countries are appropriate for tropical systems (e.g., Byamukama et al., 2000; Nshimyimana et al., 2014). For example, *E. coli* may be able to persist for some time in tropical freshwaters (Carillo et al., 1985; Jiminez et al., 1989); Rivera et al. (1988) showed that *E. coli* can be found in epiphytic bromeliads from tropical rain forests. Furthermore, and as pointed out by Winfield and Groisman (2003) it is probable that *E. coli* and other FIB can persist and even proliferate in tropical environments, particularly those with high temperatures and elevated nutrient and organic matter concentrations. Therefore, the environmental factors (**Figure 2**) that control the persistence of FIB and the microbial pathogens they are supposed to be a proxy for may well result in a large dichotomy between the survival of FIB and the pathogens.

The principal methods used for detecting FIB are culture based. In general, this method is simple and involves the inoculation of a known volume of sample into a specific culture media and then incubation of the sample for 24–48 h at 37^∘^C (or 44–45^∘^C for the thermotolerant species). The number of positive samples or the number of colonies is then counted. This method comes in many different formats (e.g., test-tubes, microplates, larger volume dip plates) which can be easily adapted to analytical capacities of the site. It is also relatively cheap, very easy to use and requires no complex equipment and is for these reasons the most widespread method in use. Moreover, several accredited methods exist providing a standardized protocol that facilitates comparison amongst laboratories. Often serial dilutions of the sample can be used to provide an estimation of the ‘most probable number’ (MPN) such as in the standardized microplate method (ISO 9308-3) as is used for determining the presence and number of *E. coli*. An alternative method involves filtering or inoculating samples onto specific culture media and then counting the number of colonies after a period of incubation, usually 24–48 h (e.g., Chahinian et al., 2012). However, these methods are all culture based and thus rely on the culturability of the bacteria present, a factor that can vary considerably as a function of the bacterial species investigated, the environmental parameters and the physiological state of the bacteria (Colwell, 2000). The other main disadvantages are that they require at least 24 h before a result can be obtained and they are non-specific and so it is difficult to identify the source of the contamination (human or animal) or even if the bacteria is actually from fecal matter and not from another source such as a native soil population (Byappanahalli et al., 2006, 2012b).

Some of the newer methods available can track the source of contamination through the use of specific biomarkers such as fecal sterols and stanols (Isobe et al., 2002; Jarde et al., 2005, 2007; Solecki et al., 2011), through the presence of host species specific (e.g., human or pig) bacteria (Jeanneau et al., 2012; Neave et al., 2014; Nshimyimana et al., 2014) or by tracking chemical contaminants that are often found in sewage such as caffeine or the pain medicine acetaminophen (Jeanneau et al., 2012; Ekklesia et al., 2015a). Knowing the source of the contamination can be particularly useful in rural, developing areas where human and livestock often live in close proximity.

Fecal sterols and stanols, such as the molecules coprostanol or sitostanol, are found in animal feces and are produced from the hydrogenation of cholesterol in the intestine. The presence or absence of these molecules can therefore be used to determine if fecal contamination is present. This method has been used as an indication of fecal contamination in both soils and water (Isobe et al., 2002, 2004; Jarde et al., 2005). Moreover, when combined with measurement of other structurally related sterols to provide a ‘species specific fingerprint,’ information on the source of the contamination can be obtained (Jaffrezic et al., 2011; Jeanneau et al., 2011). For example, the ratios of coprostanol:(coprostanol+24-ethylcoprostanol) and of sitostanol:coprostanol have been proposed as being good indicators of the origin of fecal contamination as they differ between fecal matter source (Gourmelon et al., 2010).

The use of host specific genetic markers such as HF183 that is specific to human fecal matter or Pig 2 Bac that is specific to pig fecal matter has also been proposed as a powerful way to identify the sources of fecal contamination (Mieszkin et al., 2009; Harwood et al., 2014; Neave et al., 2014; Nshimyimana et al., 2014). In this method, known as microbial source tracking (MST), molecular techniques are used to determine the presence or absence (PCR) and relative abundance (qPCR) of species-specific microbial biomarkers (Harwood et al., 2014). This method can be particularly useful if the source of fecal contamination (human or animal) needs to be identified. However, and as noted by Harwood et al. (2014) in their review on MST, often the relative abundances of the selected genetic markers are not strongly correlated with FIB or with the pathogens for which they are supposed to be a proxy. This may be due to the fact that the die-off rates of the host specific markers can differ from those of the FIB or pathogens or that the die-off rates are variable as function of the physico-chemical state of the examined environment (Jeanneau et al., 2012). Nevertheless, future developments and refinements of this method will probably lead to the resolution of this particular problem.

Similarly, chemical indicators specific to humans can be used to trace the source of contamination. However, although several candidate chemicals (e.g., caffeine or acetaminophen) have been proposed as a marker of human contamination for the moment no single chemical indicator has emerged as a good proxy for the presence of pathogens of fecal origin (Jeanneau et al., 2012; Ekklesia et al., 2015a). It is for this reason that these latter authors also proposed that chemical indicators should probably be best used as a confirmatory method to guard against false positives. This is potentially of importance in tropical systems where to the potential for naturalization and growth of FIB outside of the host is higher. A second problem with this method is related to the decay or removal times of these chemical indicators relative to the die-off rates of FIB and the water borne pathogens of fecal origin (Jeanneau et al., 2012). Indeed, these authors found that caffeine persisted longer in both seawater and freshwater than the specific biomarker HF183 or stanols. Therefore, the presence of caffeine may well indicate recent contamination. However, this work was carried out at 20^∘^C and it is unknown how persistence changes at higher temperatures such as are found in the tropics. Nevertheless, it is important to keep in mind their respective degradation rates relative to those of FIB or of water borne pathogens when using this method.

These methods all have the advantage that they are culture independent and have been shown to be appropriate for use in tropical systems (Isobe et al., 2002, 2004; Nshimyimana et al., 2014; Ekklesia et al., 2015a). However, they all require relatively high level analytical capacities, access to which can be difficult in developing countries. Nevertheless, these newer methods provide an interesting way to track microbial contamination without needing to rely on culture techniques.

## Environmental Factors

The persistence of FIB in the environment is determined by a number of factors such as the physico-chemical status of the system (changes in temperature, pH, humidity, salinity…), the presence of other bacteria, viruses and predators and the metabolic capacities of the bacteria (Ishii and Sadowsky, 2008; Byappanahalli et al., 2012a; Korajkic et al., 2013b). All of which will influence the decay (or loss) rates of FIB (Anderson et al., 2005; Byappanahalli et al., 2012a). Moreover, the ability of the microbes to compete effectively with the rest of the bacterial community for nutrients and dissolved organic matter (DOM) will also influence their ability to persist and proliferate outside of their host species (**Figure 2**).

### Temperature and Climatic Influences

Shifts in temperature can affect the persistence of FIB. Boaretti et al. (2003) showed that *E. coli* remained viable for up to 33 days after incubation at low nutrient and temperature levels. Ishii et al. (2006) found that when incubated in non-sterile soils, *E. coli* grew and multiplied at temperatures of 30 and 37^∘^C and persisted for over 1 month in soils incubated at 25^∘^C. More recently, Chahinian et al. (2012) found that FIB remained culturable after 163 days at 5^∘^C, a temperature considerably lower than that of mammalian intestines. Thus it is clear that persistence in the environment can introduce a problem when these indicator bacteria are used as their appearance may or may not indicate that pathogenic bacteria are present. Moreover, all of this work was conducted on temperate soils and sediments and, at present, much less is known about the survival of these organisms in tropical soils and water at *in situ* temperatures.

Data from temperate regions also shows a strong link between survival and season (Dutka and Kwan, 1980; Isobe et al., 2004; Oliver et al., 2006). In temperate latitudes highest rainfall occurs during the colder months thereby tending to reduce the number of FIB in surface waters (e.g., Mitch et al., 2010), but in tropical countries higher rainfall occurs in hotter months which can have consequences on both survival but also the re-inoculation of FIB to the adjacent water bodies (Pandey et al., 2012a). For example, Isobe et al. (2004) observed higher concentrations of FIB during the dry season than in the wet season in the Mekong delta, Vietnam. The authors proposed that this was due to lower dilution of polluted waters during the dry season when flow is much lower.

### Desiccation and Rehydration Cycles

Desiccation and rehydration also appear to play an important role in determining the release and subsequent transfer of FIB from soils to the water column. Soil humidity depends on a multitude of factors including the soil intrinsic properties (e.g., hydrodynamic characteristics), slope gradient, surface roughness, bioturbation, land use, and plant cover (Ribolzi et al., 2011b; Patin et al., 2012). Solo-Gabriele et al. (2000), working in southern Florida showed that during a sequence of repeated drying and rehydrating of a soil the numbers of *E. coli* drastically increased (by over a factor of 20). Moreover, the cycles of dessication-rehydration were quite short (6 h of each process). Thus it is clear that the rehydration of the soils, such as may occur during a precipitation episode, can play an important role in the survival of FIB as has been recently shown for environmental bacteria (Kaisermann et al., 2013).

### Organic Matter and Nutrients

Although FIB are considered to be enteric bacteria and therefore adapted to a nutrient and organic matter rich, low oxygen environment in their host there is some evidence that they can persist in the ecosystem and particularly so in tropical soil environments (Byappanahalli and Fujioka, 1998; Winfield and Groisman, 2003; Anderson et al., 2005; Ishii et al., 2006; Ishii and Sadowsky, 2008). How these bacteria are able to persist and become naturalized in non-host environments such as soils or sediments is related to the metabolic capacities of the bacteria and to the bioavailability of DOM present. It is probable that indigenous bacteria (i.e., of non-fecal origin) will be more adapted to the environment (i.e., outside of the host), however, this does not mean that FIB subject to sub-optimum growth requirements will disappear, it just means that they will grow less well due to competition in ecological niches that are less well-adapted to their growth requirements. Upon release from the host, FIB are faced with an environment that is often colder, more dilute and has higher oxygen concentrations, although it should be noted that sediments with low oxygen concentrations may provide a more propitious environment for FIB. Moreover, the organic matter quality and quantity are probably vastly different between the host and the external environment and it is reasonable to expect that riverine organic matter is of a much lower bioavailability than enteric organic matter. Recent work has shown that intestinal bacteria such as *E. coli* are adapted to use small carbohydrate molecules that are found in abundance in the intestines (Zoetendal et al., 2012). However, the concentrations of these highly bioavailable molecules in water are several orders lower (Benner, 2002). Fujioka and Byappanahalli (2001) have shown that FIB also have the capacity to degrade a series of carbon sources found in soils and Bouvy et al. (2008) working in Senegal found that it was substrate concentration rather than temperature that controlled FIB persistence in a coastal system subject to high sewage inputs. It is therefore probable that given adequate concentrations of bioavailable carbon and nutrient sources FIB will be able to survive and possibly even proliferate in tropical environments.

The work of Garzio-Hadzick et al. (2010) also supports this hypothesis as they have shown that the survival of *E. coli* was higher in soils with a higher organic matter content. In addition, Topp et al. (2003) found that survival was high in loamy soils and that this survival increased with the addition of manure. These authors proposed that the community composition of the microbial community, including indicator and pathogenic bacteria is probably influenced by the type and amount of organic matter in the soils. Obviously, this situation could be particularly problematic in areas where organic matter, such as fresh manure, is recursively applied (Trevisan et al., 2002). This is often the situation in developing countries where fresh manure is frequently used as an economical fertilizer option for both fields and in aquaculture (e.g., Yajima and Kurokura, 2008). In one of the few articles treating this subject in tropical soils, Byappanahalli and Fujioka (2004) have shown that microbial community structure and soil moisture content play an important role in controlling the proliferation of FIB in soils, similar to the work of Oliver et al. (2006) who worked in a temperate system. This means that in situations with adequate soil moisture contents, such as can be found in tropical, humid systems, the proliferation of FIB cannot be ruled out. This is further underlined by the fact that *E. coli* isolated from soils is generally found to be genetically distinct from *E. coli* found in host intestines (Byappanahalli et al., 2006, 2012b). This suggests that the environment selects for specific clonal attributes thus allowing the bacteria to become naturalized (De Wit and Bouvier, 2006).

### Sunlight

Sunlight inactivation of FIBs is an important process in natural waters (Sinton et al., 2002; Chan et al., 2015). Although sunlight inactivation is important in rivers and streams, it is even more important in estuarine and saline waters, probably as a consequence of the higher light penetration levels due to lower turbidity levels. Cho et al. (2010a,b) working in a South Korean creek found that sunlight during dry weather periods was the most important factor controlling FIB populations. In tropical regions with high solar radiation intensities, it is probable that sunlight is an important factor controlling the inactivation of FIB as has been already shown for indigenous bacteria in clear waters with high light penetration depths (e.g., Conan et al., 2008). Troussellier et al. (2004) working with cultures of *E. coli* isolated from a coastal system (Senegal River, Senegal) also showed that survival of the bacteria dramatically decreased at high light levels. It therefore seems that light penetration in the water column plays an important role in determining survival. We can therefore hypothesize that considering that tropical freshwater systems are often highly turbid (Milliman and Meade, 1983; Milliman, 1995) thereby reducing light penetration, and that temperature is high, survival rates will be much higher than in systems with relatively high light penetration levels. Although this remains to be rigorously tested in a full range of tropical freshwater systems, it is clear that the blind application of survival rates determined in the cold, clear waters of temperate systems may not provide an appropriate rate for highly turbid, warm tropical situations.

### Microbiota

Protozoan predation is a major biological FIB removal mechanism (Korajkic et al., 2013b; Wanjugi and Harwood, 2013). Viral lysis has also been identified as an important factor and the combined effect of grazing and viral lysis can be responsible for up to 70% of the loss rates over a 120h period (Korajkic et al., 2014). However, it appears that the rates vary with FIB species and with location. For example, Korajkic et al. (2014) found while that grazing and viral lysis resulted in a significant decrease in enterococci numbers, this was not the case for *E. coli*, at least in the site they examined (upper Mississippi River). This differential species effect poses some interesting questions on the response of FIB to grazing and viral lysis, particularly as it is in contrast to other work that showed a significant impact of microbiota on FIB abundance (Menon et al., 2003; Wanjugi and Harwood, 2013). Moreover, the differences between this results of this work and that of previous work from sub-tropical waters by the same authors (Korajkic et al., 2013a) points toward a potentially significant role of microbiota in other tropical systems. It should borne in mind that grazing of FIB may not result in the immediate death of the cell as it has been shown that FIB can pass through the digestive tract of predators and still be viable. For example, Smith et al. (2012) found that *E. coli* remained culturable even after passage through the aquatic ciliate *Tetrahymena* sp. in a laboratory. How this result can be transposed to a natural, tropical environment remains to be evaluated, nevertheless, this process should be kept in mind.

### Associations with Particles and other Non-Host Organisms

Many pathogens are known to produce and to proliferate in biofilms (September et al., 2007; Wingender and Flemming, 2011). Biofilms are thin films composed carbohydrates produced by biological processes such as photosynthesis or bacterial activity (Burmolle et al., 2012). Biofilms not only provide bacteria with an organic matter matrix of a higher concentration than in the water column, it also can provide access to nutrients as well as providing a type of “glue” that will allow the bacteria to remain associated with a particle. In soils and sediments, bacteria, including FIB and pathogens, tend to be associated with particles as opposed to in the free-state (e.g., Oliver et al., 2007). In aquatic systems the percentages of particle associated bacteria are highly variable and can range from 10% in clear waters with very low organic particle loads to over 70% in estuaries with high particle loads (Crump et al., 1998; Lemee et al., 2002). Moreover, Suter et al. (2011) have shown that higher proportions of FIB are associated with particles (52.9 ± 20.9% and to over 90% in some areas) than total bacteria (23.8 ± 15.0%) and that, perhaps unsurprisingly, that these values were related to turbidity levels.

The attachment of FIB and non-FIB bacteria to particles is also due to changes in ionic strength of the media (Zita and Hermansson, 1994; Otto et al., 1999). The dilution of stream water or pond water by rain will alter the ionic strength thereby influencing the attachment or detachment of bacteria to particles through influencing the electrostatic properties and the conformation of biopolymers. Indeed, it is known that the physico-chemical strength of the substrate surface and the specific exo-polymeric components of the bacterial surface itself can play an important role in determining if a bacteria becomes attached to a particle (Regina et al., 2014).

In one of the few articles treating the factors controlling FIB concentrations in high altitude tropical countries, Byamukama et al. (2005) show that the presence of FIB in aquatic systems was correlated to total suspended solids (TSSs) concentrations. In rivers and streams, turbidity, which is directly related to the concentration of particles in the water column, tends to be higher in tropical regions due to the highly erosive nature of precipitation, basin geomorphology, land use and soils and sediment erodibility in the watersheds (Milliman and Meade, 1983; Milliman and Syvitski, 1992). It is therefore probable that higher proportions of FIB will be particle attached in these ecosystems. However, at the moment, little or no information exists on the proportions of attached and free FIB in tropical water bodies.

*E. coli* can also survive in periphyton communities in streams. Periphyton includes a wide diversity of organisms that live in the biofilms attached to surfaces in aquatic systems. This community includes diatoms and other phytoplankton, protozoa, environmental bacteria and other microorganisms. Ksoll et al. (2007), working in Lake Superior showed that FIB can persist and even proliferate in the periphyton community. Moreover, these authors also proposed that some of the *E. coli* strains found had become naturalized to the periphyton community thus allowing them to become a self-sustaining population.

Work in temperate and sub-tropical sites has also revealed the presence of FIB associated with macrophytes and filamentous cyanobacteria (Byappanahalli et al., 2003; Vijayavel et al., 2013). Karim et al. (2004) working in a constructed wetland in Arizona reported that the presence of macrophytes increased the persistence of FIB in the water column and sediments. They also found that die-off rates were much lower in the sediments than in the water column, pointing toward a potentially important role of particles in maintaining FIB populations viable. To our knowledge, little published information exists on the presence of FIB in periphyton or in association with macrophytes or cyanobacteria in tropical, humid, systems.

### Storm Events and Inter-Storm Flow Periods

Epidemiological studies show a statistical relationship between diarrhea and climate variables (Jagai et al., 2009; Nichols et al., 2009). Recently, in a review of the probable future impacts of climate change on pathogens in surface waters, Hofstra (2011) pointed out that future shifts in climate will only increase the spread of waterborne pathogens. This will be even more problematic in developing countries where infrastructures to deal with floods and adequate public health protection measures are lacking. Hrdinka et al. (2012), working in the Czech Republic compared the impacts of floods and droughts on water quality and they found that flood events had a significantly greater impact on water quality than periods of low flow. This was due to the washing out of contaminants during flood events. In Bangladesh, Kunii et al. (2002) also demonstrated that flooding drastically reduced the health of the communities affected. However, the rigorous evaluation of this relationship in other tropical regions is hampered by the availability of epidemiological data (Karanis et al., 2007).

The processes responsible for the dispersion of FIB depend on the spatial scale of observation and given the importance of water in the dissemination of FIB, it is perhaps not surprising that hydrology plays an integral role (**Figure 2**). Having an accurate description of how water (e.g., rain) and the particles and solutes carried by that water are transferred from land to water is still a fundamental hydrological challenge (Klaus and McDonnell, 2013). Yet this is a prerequisite for understanding the spread and fate of FIB. The relative amount of groundwater (i.e., with little or no load of particles and FIB) controls the magnitude of the dilution effect of bacteria loads during floods. This is in contrast to overland flow which strongly contributes to soil erosion and hence, FIB erosion processes (Ribolzi et al., 2011a). Hence, floods dominated by overland flow will be associated with higher loads of suspended particles and probably FIB. Once delivered to the river, sediment and FIB can then accumulate on riverbeds before being re-suspended after an increase in river discharge (Navratil et al., 2010).

Working in a highly urbanized watershed in South Korea (temperate climate with four distinct seasons), Cho et al. (2010a,b) found that FIB populations significantly increased during precipitation, with greater concentrations occurring at higher rainfall intensity. During dry weather, FIB populations decreased in the presence of sunlight but quickly recovered at night due to continuous point-source inputs. Ekklesia et al. (2015b) who worked in tropical, urban Singapore also highlighted the importance of diurnal patterns of water flow on FIB populations. The authors found a considerable difference in FIB levels over a 24 h period and underlined the importance of selecting an appropriate sampling time if representative contamination levels are to found. Sinclair et al. (2009) and Stumpf et al. (2010) also observed that the concentrations of FIB in storm waters are higher than baseflow loads. Moreover, Stumpf et al. (2010) found that stormwater runoff can account for a much greater portion of overall FIB loading in creeks as compared to non-event baseflow loading. Working in tidal creek headwaters in North Carolina, USA these authors showed that total storm loading, averaged across all storms, was as much as 30 and 37 times greater than baseflow loading for *E. coli* and *Enterococcus* spp., respectively. They found that FIB were weakly correlated with TSS and strongly correlated with flow rate and the different stages of the hydrograph. However, their pollutographs indicated a large intra-storm variability in FIB. They also noted that the increases in FIB loading during stormflow events were due to fecal contamination from stream beds as well as from terrestrially associated fecal contamination that is scoured from land and transported via stormwater into receiving waters. Strauch et al. (2014) recently examined the impact of mean annual rainfall and short term rain events on FIB contamination in a series of upland catchments (over 600 m above sea level) in Hawaii. They found short term rain events had a higher impact of FIB concentrations in low annual rainfall areas than in high annual rainfall areas. They also observed that declines in forest cover and increases in urbanization lead to increased FIB concentrations.

In tropical systems, the relationship between baseflow and stormflow can also play an important role in the erosion and resuspension of sediments (Gourdin et al., 2014). Tropical systems are often characterized by heavy rainfall that can be highly erosive (Lal, 1983). Erosive rainfall events result in strong overland flow events which can accelerate detachment processes and increase the numbers of particles and of particle attached bacteria. This is exacerbated during exceptional events, such as storms, and during river spates when the transfer of highly contaminated water to downstream areas occurs (Ribolzi et al., 2011a). Highly erosive rains also increase the splash effect, where by rain droplets with high kinetic energies increase erosion and thereby increase the export of particles and bacteria to downstream systems (Planchon et al., 2000). This results in the resuspension of particles as a function of flow. Although some work has looked at the relationship between flow and particle resuspension (Pandey et al., 2012b; McDaniel et al., 2013; Pandey and Soupir, 2013), little or no work have been done on this aspect in tropical systems where turbidity is elevated. The links between particles and FIB in small, upland streams where contamination levels and particle concentrations can be high (Milliman and Syvitski, 1992) have also been largely ignored. This is despite the importance of streambeds as a source of *E. coli* as was underlined by Kim et al. (2010) who monitored and modeled bacterial release from a streambed with the modified SWAT (Soil and Water Assessment Tool) model.

## Impact of Land-Use

Other than human waste, agriculture is the other major source of FIB in aquatic systems and land use is known to play an important role in the inoculation, persistence and dissemination of FIB (Zhang et al., 2012). Agricultural practices in tropical systems often differ substantially from those of temperate ecosystems. Temperate systems are generally characterized by an industrialized agriculture with large scale farms and intensive production practices. This is in contrast to the situation in tropical regions where agriculture is often based on subsistence farming and extensive production practices. Farmlands are also often located on steeply sloping lands in the upper parts of catchments.

The export of matter from soils to an adjacent aquatic ecosystem is controlled, in part, by the type of plant cover present, the slope and the concentration of organic matter in the soils (Magdoff and Weil, 2004; Podwojewski et al., 2008; Soupir and Mostaghimi, 2011; Janeau et al., 2014; Rochelle-Newall et al., 2014). Moreover, the export of organic matter and bacteria can be particularly high in sloping lands both of which can affect downstream aquatic ecosystems (Ribolzi et al., 2011b; Pommier et al., 2014). The impacts of agriculture on the ecosystem are therefore not expected to be the same in temperate and tropical systems. Despite this, although much work has examined the impact of land cover and agricultural practice on the export of FIB from fields to aquatic systems in temperate systems (e.g., Crowther et al., 2003; Pachepsky et al., 2006, 2011), the processes affecting the transport of FIB from agricultural sources in tropical and sub-tropical systems are still poorly understood.

In intensive farming areas where manure production is high there is a higher possibility of soil and aquatic contamination (Brown et al., 2014; Pandey et al., 2014). Moreover, if these production zones are near to streams and rivers or if the animals have direct access to the stream then contamination of the water course by FIB will be highly probable (e.g., Crowther et al., 2003; Gannon et al., 2005; Vidon et al., 2008a; Monaghan et al., 2009). Kloot (2007) examined *E. coli* contamination in a rural South Carolina watershed and found that headstreams with high numbers of cattle in the adjacent fields had the highest FIB densities. In the American Midwest, Vidon et al. (2008a) found that during periods of low flow (summer and fall) cattle access to streams could drastically impact the bacterial water quality. They also highlighted the importance of good management practice in reducing the contamination levels in the streams. Monaghan et al. (2009), working in Southern New Zealand, described some key linkages between land management activities and stream water quality. These authors proposed that limiting stock access to unfenced lengths of a stream and the planting and maintaining of riparian margins were important land management practices. The role of good management practice in reducing contamination from agricultural activities was further underlined by Gannon et al. (2005) in Southern Alberta, Canada. These authors proposed that the use of in-stream reservoirs may significantly reduce contamination thereby increasing the quality of limited rural water supplies sufficiently that their reuse and safe discharge into downstream water sources was possible.

In tropical areas, humid zones are often characterized by rice farming and small scale fish pond farming that are sometimes subject to fertilization by fresh animal manure (e.g., Yajima and Kurokura, 2008). This practice can result in high levels of FIB contamination in ponds and in downstream waters, potentially impacting health of local communities. Riparian zones are also known to play an important role in trapping sediments (Ziegler et al., 2006; Vigiak et al., 2008; Wanyama et al., 2012) and bacteria in both tropical and temperate systems subject to agricultural inputs (Monaghan et al., 2009; Ribolzi et al., 2011a). However, in many areas riparian zones are increasingly used for cultivation and so it is probable that the levels of transport of FIB from the terrestrial system to the aquatic environment will only increase. Wetland areas are also well-known as trapping zones and in temperate systems much work has been done on the retention and persistence of FIB in sediments in freshwater, estuarine and coastal systems (e.g., Chu et al., 2011; Pachepsky and Shelton, 2011; Chahinian et al., 2012). However, to our knowledge, with the notable exception of the work of Ribolzi et al. (2011a) who worked in an upland agricultural catchment in Laos, little other published data presently exists on the role of riparian vegetation or wetlands in the retention of FIB in tropical systems.

Another factor that needs to be taken into account is the application of agrochemicals such as pesticides, herbicides, and antibiotics. Through their selective effects on different soil and aquatic organisms they affect survival of FIB and the associated pathogens (Staley et al., 2014). The reduction of protozoan grazing and the lessening of bacterial competition through selective inhibition were found to be the main mechanisms via which agrochemicals indirectly increase FIB concentrations. In contrast, no direct impact was observed on FIB populations (Staley et al., 2011, 2012). This work was conducted in small incubations in the laboratory in the US and for the moment the impact of agrochemicals on FIB survival *in situ* in other environments has yet to be examined. Given the increasing application of agrochemicals in tropical, developing areas we can ask how FIB in these warmer, more humid systems might respond to the additions of agrochemicals. Clearly, this is an area of research that should be investigated because if the same indirect effects are observed in the tropics the increasing use of agrochemicals may well-exacerbate the problems of FIB contamination in aquatic systems.

Tropical, rural areas are often characterized by the close proximity of human habitations without adequate waste treatment systems to streams and rivers. This nearness of the primary source of FIB (e.g., human and animal waste; **Figure 2**) and the stream network constitutes an elevated risk factor in terms of contamination. Indeed, many villagers use the water source for multiple uses such as a potable water supply, for bathing and washing and for evacuation of waste (**Figure 1**). Clearly this situation is problematic for the downstream users of the water resource, particularly as in many rural areas no water treatment systems are in place for drinking water or for wastewater treatment. Nor are there surveillance networks in place to detect and alert the population at times of high contamination risk.

## Modeling

Under future global and land use change scenarios, it is anticipated that tropical systems will be subject to shifts in the frequency and intensity of extreme rainfall events. This, combined with an intensification of agriculture, will potentially induce drastic changes in overland flow, soil erosion (e.g., Thothong et al., 2011) and, in turn, in the transport of FIB to aquatic systems (Hofstra, 2011). One way of addressing this challenge is to use mathematical models to investigate the effects of land use and climate change on the hydrology and transport of FIB with the objective of reducing the risks to humans (Miller et al., 2013).

Several models have been developed to model and predict the distribution of FIB in streams and rivers. In general, three main types of approach are used. The first are statistical-based models such as multiple linear regression and artificial neural networks (e.g., Thoe et al., 2012). This type of model uses input variables, usually environmental parameters, to provide an output, usually FIB concentration, via a series of multivariate linear regressions (Mahloch, 1974; Chu et al., 2011). The main advantage of this type of model is its facility and rapidity of implementation. However, the principal disadvantages are that this type of model is site specific and is based on statistical relationships, meaning that none of the associated physical and biological processes are explicitly taken in account.

The second type corresponds to the mechanistic (or physical) models that are process based and that describe, for example, the transport of FIB using one-dimensional advection dispersion equations (Wilkinson et al., 1995). More recent models of this second type also take into account the resuspension of streambed sediments and their associated FIB concentrations (e.g., Cho et al., 2010b). Contrary to the first group, this group of models permits the analysis and ranking of the underlying mechanisms responsible for the mobilization, transfer and concentration of FIB in the system. However, as for all physical models, the model needs to be parameterized and requires data inputs that are not always available, particularly so in tropical or developing countries with little or no monitoring networks.

The third group are the watershed scale, spatially distributed and semi-distributed models such as SWAT (Arnold et al., 1993) or the SENEQUE/Riverstrahler (Billen et al., 1994). These models take into account watershed morphology, spatial distribution of soil properties and land use, point sources of pollution as well as hydrology using a simplified semi-physical or conceptual approach. This approach allows the development of complex scenarios of the impacts of changes in climate and human activities on river water quality in the watershed. These models can also take into account resuspension of streambed bacteria (Kim et al., 2010), FIB die-off rates, in some cases, the role of solar radiation in influencing these die-off rates (Cho et al., 2012). This type of model provides an interesting way of looking at how point and diffuse sources in the watershed alter the river transport of particles including FIB and solutes (Lee et al., 2001; Pachepsky et al., 2006; de Brauwere et al., 2014). The advantage of this latter type of model is that it can be used to predict the impact of future changes in the system and their potential impacts on public health on the catchment scale (Kashefipour et al., 2002; Cho et al., 2012). The application of this type of model to study FIB transport and fate therefore provides some interesting avenues of future research in tropical systems.

Each of these models requires knowledge of the distribution, behavior and ecology of FIB and the factors determining their load. Moreover, for the second and third types, comprehensive knowledge of solutes and particles over a relatively long period of time as well as knowledge of the drainage basin morphology and climate is essential. This may well prove difficult or even impossible in some areas where no data presently exists. Already recognized as a problem in temperate systems (Crowther et al., 2003), this becomes even more critical in tropical systems for which little data exists on region specific FIB die-off, settling, storm wash-off and resuspension rates, all key processes in controlling FIB levels. Moreover, and as pointed out by Cho et al. (2012), an understanding of these key processes is critical for the adequate implementation of FIB management and mitigation plans in the natural environment. Indeed, although some of the existing models have been adapted to tropical systems (e.g., Le et al., 2005; Luu et al., 2010), few, to our knowledge, have extended their work to include the transport and fate of FIB in these systems. A notable exception to this is the work from the coastal area of sub-tropical Hong Kong (Thoe et al., 2012). Clearly this is an area that merits future study.

## Conclusion

The developing countries of the intertropical belt will be the worst hit by global change (Gross, 2002). This region already has the highest global soil erosion rates (Lal, 1983; Milliman and Meade, 1983) and it is anticipated that these rates will increase in the future given the anticipated changes in global climate. The high erosion rates found in these regions will further favor the development of sediment deposition and resuspension zones and with them, potential reservoirs for FIB. A conceptual diagram of the processes affecting FIB in tropical systems is given in **Figure 2**. The primary sources of FIB in the tropics (humans and livestock) all contribute to the dissemination of FIB at the soil surface in cultivated lands and inhabited areas via open air defecation and latrines, manure application and livestock. The primary sources also directly contaminate adjacent aquatic ecosystems via direct waste and wastewater release. In humid, tropical zones rain events are often characterized by high intensities and depth. During these events, contaminated soils are washed off in overland flow that contains high suspended sediment loads (Gourdin et al., 2014). This overland flow in turn contaminates off-site soils and downstream water bodies resulting in a secondary reservoir of FIB. This secondary reservoir also includes cultivated land and inhabited rural areas, has a hypothesized lower FIB survival rate than that of the primary source. This lower survival is due to in part to the higher light and temperatures in the tropics and their interactive effects. The tertiary source, comprised of stream bed sediments and hyporheic zones, offers conditions that are more similar to the host intestinal tract: low or no light levels due to the density of riparian vegetation, low oxygen conditions and reduced predation by protozoans (Pachepsky and Shelton, 2011). It should be noted that the reduction in predation by protozoa in sediments is probably not specific to the tropics. However, it may well be that the sedimentary dynamics in these zones tend to accentuate the phenomena of erosion (Burmolle et al., 2012) and deposition in the rivers and streams (e.g., Gourdin et al., 2014) and thus multiply the number of potential reservoirs of these tertiary sources. This is all the more probable considering that the particles washed off from the soil surface are often rich in organic carbon (Gourdin et al., 2015). All of these physical, biological and ecological factors probably combine to favor the survival of FIB outside of their host. Moreover, in tropical humid environments, this is further compounded by higher and more stable temperatures, higher nutrient and organic matter availabilities and higher annual rainfall. All of which interact to favor the persistence and potentially the proliferation of FIB in this tertiary source (**Figure 2**).

## Future Directions

Although much is known about the sources, transport and fate of FIB in temperate climates it is clear that a more detailed investigation of the factors controlling the environmental persistence of FIBs in tropical environments is needed. The majority of data on the concentration, dispersal, persistence, or proliferation of FIBs comes from studies conducted in either temperate systems or in tropical systems in developed countries and little or no data is available on these parameters in developing nations where the death and disability rates due to the use of unclean water are highest. This paucity of information on tropical ecosystems, with the exception of the pioneering work of conducted in Hawaii, Uganda and Singapore (e.g., Byappanahalli and Fujioka, 1998; Byamukama et al., 2005; Nshimyimana et al., 2014; Ekklesia et al., 2015a) means that much remains unknown.

For example, we do not know anything about the survival of FIB or indeed of the pathogens they are supposed to be a proxy for, in tropical upland soils, nor on their transport downstream in small agricultural watersheds subject to monsoonal weather patterns. Nor do we have an idea of how the rates of contamination under baseflow and stormflow will change their transport in the future. Given the importance of land use in the export of soil and the associated microbial pathogens, it is probable that better management of the landscape mosaic and the optimization of land use will diminish the risks of pathogen survival and dissemination. However, little information exists on the efficacy of this proposition or on its applicability to tropical systems.

We do not know how the interactions between fertilizers, temperature and organic matter contents of soils impact FIB and pathogen persistence in a wide range of tropical systems, nor do we know how this persistence impacts their eventual transfer into adjacent rivers and streams. Similarly, we have no information on how agrochemicals affect FIB survival in tropical environments. We have no information on the survival or naturalization of the FIB in tropical streams and rivers and little is known on whether FIB and the pathogens they are supposed to be a proxy for, are harbored in macrophytes or in association with other phototrophs such as cyanobacteria and periphyton in stream biofilms. We have little or no information on the die-off rates of FIB in turbid, tropical systems, nor on the interactions between temperature, light and on protozoan grazing and viral lysis on their survival. These aspects need to be addressed before adequate validation data sets can be produced for the development of appropriate mechanistic models of FIB transport and fate in aquatic systems.

The development and application of statistical and watershed scale, spatially distributed models to tropical watersheds will also provide new opportunities to explore the impact of climate change on FIB and pathogen concentrations. The application of models such as SWAT may well provide an interesting avenue for this. Models that enable the testing of more realistic scenarios that include changes in all input variables are also needed. Moreover, given the importance of overland flow in determining the levels of contamination, it is critical that the contributions of groundwater and surface water are adequately taken into account. Another aspect that needs to be considered is the impact of nutrient and organic matter concentrations and quality in determining FIB survival. At present none of the models take these factors into account and this is true for temperate systems where extensive datasets exist as well as in tropical environments where it is even more critical. Given that FIB may well be able to proliferate in tropical systems, it is clear that this must be taken into account in any model that wishes to simulate FIB dissemination or proliferation.

The compilation of a comprehensive data base of epidemiological parameters will also provide a new avenue for testing the role of climate in the dispersion of FIB and the impacts of contaminated waters on public health in developing countries. More research is needed in these areas if we are to meet the Millennium Development goals as regards clean water access and the prevention of water borne diseases in these often poor, rural tropical areas. Finally, we have focused on the environmental and ecological implications of FIB and have not taken into account the socio-economic specificities of developing countries. Clearly if any progress is to be made in reducing the risks to the local populations, these factors also need to be taken into account.

## Conflict of Interest Statement

The authors declare that the research was conducted in the absence of any commercial or financial relationships that could be construed as a potential conflict of interest.

## References

[B1] Anderson K. L. Whitlock J. E. Harwood V. J. (2005). Persistence and differential survival of fecal indicator bacteria in subtropical waters and sediments. *Appl. Environ. Microbiol.* 71 3041–3048 10.1128/AEM.71.6.3041-3048.2005 15933000 PMC1151827

[B2] Arnold J. G. Allen P. M. Bernhardt G. (1993). A comprehensive surface groundwater flow model. *J. Hydrol.* 142 47–69 10.1016/0022-1694(93)90004-S

[B3] Ashbolt N. J. (2004). Microbial contamination of drinking water and disease outcomes in developing regions. *Toxicology* 198 229–238 10.1016/j.tox.2004.01.030 15138046 PMC7126529

[B4] Bain R. Cronk R. Hossain R. Bonjour S. Onda K. Wright J. (2014a). Global assessment of exposure to faecal contamination through drinking water based on a systematic review. *Trop. Med. Int. Health* 19 917–927 10.1111/tmi.12334 24811893 PMC4255778

[B5] Bain R. Cronk R. Wright J. Yang H. Slaymaker T. Bartram J. (2014b). Fecal contamination of drinking-water in low- and middle-income countries: a systematic review and meta-analysis. *PLoS Med.* 11:e1001644 10.1371/journal.pmed.1001644 PMC4011876 24800926

[B6] Benner R. (2002). “Chemical composition and reactivity,” in *Biogeochemistry of Marine Dissolved Organic Matter* eds Hansell D. Carlson C. A. (San Diego, CA: Academic Press) 59–90 10.1016/B978-012323841-2/50005-1

[B7] Billen G. Garnier J. Hanset P. (1994). Modeling phytoplankton development in whole drainage networks - the Riverstrahler model applied to the Seine River system. *Hydrobiologia* 289 119–137 10.1007/BF00007414

[B8] Boaretti M. Del Mar Lleò M. Bonato B. Signoretto C. Canepari P. (2003). Involvement of rpoS in the survival of *Escherichia coli* in the viable but non-culturable state. *Environ. Microbiol.* 5 986–996 10.1046/j.1462-2920.2003.00497.x 14510852

[B9] Bouvy M. Briand E. Boup M. M. Got P. Leboulanger C. Bettarel Y. (2008). Effects of sewage discharges on microbial components in tropical coastal waters (Senegal, West Africa). *Mar. Freshw. Res.* 59 614–626 10.1071/MF07244

[B10] Brown S. B. Ikenberry C. D. Soupir M. L. Bisinger J. Russell J. R. (2014). Predicting time cattle spend in streams to quantify direct deposition of manure. *Appl. Eng. Agric.* 30 187–195.

[B11] Burmolle M. Kjoller A. Sorensen S. J. (2012). “An invisible workforce: biofilms in the soil,” in *Microbial Biofilm: Current Research and Applications* eds Lear G. Lewis G. D. (Norfolk, VA: Caister Academic Press) 61–71.

[B12] Byamukama D. Kansiime F. Mach R. L. Farnleitner A. H. (2000). Determination of *Escherichia coli* contamination with chromocult coliform agar showed a high level of discrimination efficiency for differing fecal pollution levels in tropical waters of Kampala, Uganda. *Appl. Environ. Microbiol.* 66 864–868 10.1128/AEM.66.2.864-868.2000 10653767 PMC91912

[B13] Byamukama D. Mach R. L. Kansiime F. Manafi M. Farnleitner A. H. (2005). Discrimination efficacy of fecal pollution detection in different aquatic habitats of a high-altitude tropical country, using presumptive coliforms, *Escherichia coli*, and *Clostridium perfringens* spores. *Appl. Environ. Microbiol.* 71 65–71 10.1128/AEM.71.1.65-71.2005 15640171 PMC544213

[B14] Byappanahalli M. N. Fujioka R. S. (1998). Evidence that tropical soil environment can support the growth of *Escherichia coli*. *Water Sci. Technol.* 38 171–174 10.1016/S0273-1223(98)00820-8

[B15] Byappanahalli M. N. Fujioka R. S. (2004). Indigenous soil bacteria and low moisture may limit but allow faecal bacteria to multiply and become a minor population in tropical soils. *Water Sci. Technol.* 50 27–32. 15318482

[B16] Byappanahalli M. N. Nevers M. B. Korajkic A. Staley Z. R. Harwood V. J. (2012a). Enterococci in the environment. *Microbiol. Mol. Biol. Rev.* 76 685–706 10.1128/MMBR.00023-12 23204362 PMC3510518

[B17] Byappanahalli M. N. Yan T. Hamilton M. J. Ishii S. Fujioka R. S. Whitman R. L. (2012b). The population structure of *Escherichia coli* isolated from subtropical and temperate soils. *Sci. Total Environ.* 417 273–279 10.1016/j.scitotenv.2011.12.041 22264918

[B18] Byappanahalli M. N. Shively D. A. Nevers M. B. Sadowsky M. J. Whitman R. L. (2003). Growth and survival of *Escherichia coli* and enterococci populations in the macro-alga *Cladophora* (Chlorophyta). *FEMS Microbiol. Ecol.* 46 203–211 10.1016/S0168-6496(03)00214-9 19719574

[B19] Byappanahalli M. N. Whitman R. L. Shively D. A. Sadowsky M. J. Ishii S. (2006). Population structure, persistence, and seasonality of autochthonous *Escherichia coli* in temperate, coastal forest soil from a Great Lakes watershed. *Environ. Microbiol.* 8 504–513 10.1111/j.1462-2920.2005.00916.x 16478456

[B20] Carillo M. Estrada E. Hazen T. C. (1985). Survival and enumeration of the fecal indicators *Bifidobacterium adolescentis* and *Escherichia coli* in a tropical rain forest watershed. *Appl. Environ. Microbiol.* 50 468–476. 3901921 10.1128/aem.50.2.468-476.1985 PMC238644

[B21] Chahinian N. Bancon-Montigny C. Caro A. Got P. Perrin J. L. Rosain D. (2012). The role of river sediments in contamination storage downstream of a waste water treatment plant in low flow conditions: organotins, faecal indicator bacteria and nutrients. *Estuar. Coast. Shelf. Sci.* 114 70–81 10.1016/j.ecss.2011.09.007

[B22] Chan Y. M. Thoe W. Lee J. H. W. (2015). Field and laboratory studies of *Escherichia coli* decay rate in subtropical coastal water. *J. Hydro Environ. Res.* 9 1–14 10.1016/j.jher.2014.08.002

[B23] Cho K. H. Cha S. M. Kang J.-H. Lee S. W. Park Y. Kim J.-W. (2010a). Meteorological effects on the levels of fecal indicator bacteria in an urban stream: a modeling approach. *Water Res.* 44 2189–2202 10.1016/j.watres.2009.12.051 20138642

[B24] Cho K. H. Pachepsky Y. A. Kim J. H. Guber A. K. Shelton D. R. Rowland R. (2010b). Release of *Escherichia coli* from the bottom sediment in a first-order creek: experiment and reach-specific modeling. *J. Hydrol.* 391 322–332 10.1016/j.jhydrol.2010.07.033

[B25] Cho K. H. Pachepsky Y. A. Kim J. H. Kim J. W. Park M. H. (2012). The modified SWAT model for predicting fecal coliforms in the Wachusett Reservoir Watershed, USA. *Water Res.* 46 4750–4760 10.1016/j.watres.2012.05.057 22784807

[B26] Christenson E. Bain R. Wright J. Aondoakaa S. Hossain R. Bartram J. (2014). Examining the influence of urban definition when assessing relative safety of drinking-water in Nigeria. *Sci. Total Environ.* 490 301–312 10.1016/j.scitotenv.2014.05.010 24858228

[B27] Chu Y. Salles C. Tournoud M. G. Got P. Troussellier M. Rodier C. (2011). Faecal bacterial loads during flood events in Northwestern Mediterranean coastal rivers. *J. Hydrol.* 405 501–511 10.1016/j.jhydrol.2011.05.047

[B28] Colwell R. (2000). “Bacterial death revisited,” in *Nonculturable Microorganisms in the Environment* eds Colwell R. Grimes D. J. (Washington, DC: ASM Press), 325–342 10.1007/978-1-4757-0271-2_18

[B29] Conan P. Joux F. Torreton J. P. Pujo-Pay M. Douki T. Rochelle-Newall E. (2008). Effect of solar ultraviolet radiation on bacterio- and phytoplankton activity in a large coral reef lagoon (southwest New Caledonia). *Aquat. Microb. Ecol.* 52 83–98 10.3354/ame01204

[B30] Crowther J. Wyer M. D. Bradford M. Kay D. Francis C. A. (2003). Modelling faecal indicator concentrations in large rural catchments using land use and topographic data. *J. Appl. Microbiol.* 94 962–973 10.1046/j.1365-2672.2003.01877.x 12752803

[B31] Crump B. C. Baross J. A. Simenstad C. A. (1998). Dominance of particle-attached bacteria in the Columbia River estuary, USA. *Aquat. Microb. Ecol.* 14 7–18 10.3354/ame014007

[B32] de Brauwere A. Gourgue O. De Brye B. Servais P. Ouattara N. K. Deleersnijder E. (2014). Integrated modelling of faecal contamination in a densely populated river-sea continuum (Scheldt River and Estuary). *Sci. Total Environ.* 468 31–45 10.1016/j.scitotenv.2013.08.019 23999159

[B33] De Wit R. Bouvier T. (2006). ‘Everything is everywhere, but, the environment selects’; what did Baas Becking and Beijerinck really say? *Environ. Microbiol.* 8 755–758 10.1111/j.1462-2920.2006.01017.x 16584487

[B34] Dutka B. J. Kwan K. K. (1980). Bacterial die-off and stream transport studies. *Water Res.* 14 909–915 10.1016/0043-1354(80)90273-0

[B35] Ekklesia E. Shanahan P. Chua L. H. C. Eikaas H. S. (2015a). Associations of chemical tracers and faecal indicator bacteria in a tropical urban catchment. *Water Res.* 75 270–281 10.1016/j.watres.2015.02.037 25770447

[B36] Ekklesia E. Shanahan P. Chua L. H. C. Eikaas H. S. (2015b). Temporal variation of faecal indicator bacteria in tropical urban storm drains. *Water Res.* 68 171–181 10.1016/j.watres.2014.09.049 25462726

[B37] Ferguson D. Signoretto C. (2011). “Environmental persistence and naturalization of fecal indicator organisms,” in *Microbial Source Tracking: Methods, Applications, and Case Studies* eds Hagedorn C. Blanch A. R. Harwood V. J. (New York, NY: Springer) 379–397 10.1007/978-1-4419-9386-1_17

[B38] Fierer N. Jackson R. B. (2006). The diversity and biogeography of soil bacterial communities. *Proc. Natl. Acad. Sci. U.S.A.* 103 626–631 10.1073/pnas.0507535103 16407148 PMC1334650

[B39] Fujioka R. Byappanahalli M. (2001). “Microbial ecology controls the establishment of fecal bacteria in tropical soil environment,” in *Advances in Water and Wastewater Treatment Technology: Molecular Technology, Nutrient Removal, Sludge Reduction and Environmental Health* eds Matsuo T. Hanaki K. Takizawa S. Satoh H. (Amsterdam: Elsevier) 273–283.

[B40] Gannon V. P. J. Duke G. D. Thomas J. E. Vanleeuwen J. Byrne J. Johnson D. (2005). Use of in-stream reservoirs to reduce bacterial contamination of rural watersheds. *Sci. Total Environ.* 348 19–31 10.1016/j.scitotenv.2004.12.076 16162311

[B41] Garzio-Hadzick A. Shelton D. R. Hill R. L. Pachepsky Y. A. Guber A. K. Rowland R. (2010). Survival of manure-borne *E. coil* in streambed sediment: effects of temperature and sediment properties. *Water Res.* 44 2753–2762 10.1016/j.watres.2010.02.011 20219232

[B42] Gourdin E. Evrard O. Huon S. Lefèvre I. Ribolzi O. Reyss J.-L. (2014). Suspended sediment dynamics in a Southeast Asian mountainous catchment: combining river monitoring and fallout radionuclide tracers. *J. Hydrol.* 519B 1811–1823 10.1016/j.jhydrol.2014.09.056

[B43] Gourdin E. Huon S. Evrard O. Ribolzi O. Bariac T. Sengtaheuanghoung O. (2015). Sources and export of particle-borne organic matter during a monsoon flood in a catchment of northern Laos. *Biogeosciences* 12 1073–1089 10.5194/bg-12-1073-2015

[B44] Gourmelon M. Caprais M. P. Mieszkin S. Marti R. Wery N. Jarde E. (2010). Development of microbial and chemical MST tools to identify the origin of the faecal pollution in bathing and shellfish harvesting waters in France. *Water Res.* 44 4812–4824 10.1016/j.watres.2010.07.061 20709349

[B45] Gross J. (2002). The severe impact of climate change on developing countries. *Med. Glob. Surviv.* 7 96–100.

[B46] Guernier V. Hochberg M. E. Guegan J. F. O. (2004). Ecology drives the worldwide distribution of human diseases. *PLoS Biol.* 2:e186 10.1371/journal.pbio.0020186 PMC423130 15208708

[B47] Harwood V. J. Staley C. Badgley B. D. Borges K. Korajkic A. (2014). Microbial source tracking markers for detection of fecal contamination in environmental waters: relationships between pathogens and human health outcomes. *FEMS Microbiol. Rev.* 38 1–40 10.1111/1574-6976.12031 23815638

[B48] Hofstra N. (2011). Quantifying the impact of climate change on enteric waterborne pathogen concentrations in surface water. *Curr. Opin. Environ. Sustain.* 3 471–479 10.1016/j.cosust.2011.10.006

[B49] Hrdinka T. Novický O. Hanslík E. Rieder M. (2012). Possible impacts of floods and droughts on water quality. *J. Hydro Environ. Res.* 6 145–150 10.1016/j.jher.2012.01.008

[B50] Ishii S. Ksoll W. B. Hicks R. E. Sadowsky M. J. (2006). Presence and growth of naturalized *Escherichia coli* in temperate soils from lake superior watersheds. *Appl. Environ. Microbiol.* 72 612–621 10.1128/AEM.72.1.612-621.2006 16391098 PMC1352292

[B51] Ishii S. Sadowsky M. J. (2008). Escherichia coli in the environment: implications for water quality and human health. *Microbes Environ.* 23 101–108 10.1264/jsme2.23.101 21558695

[B52] Isobe K. O. Tarao M. Chiem N. H. Minh L. Y. Takada H. (2004). Effect of environmental factors on the relationship between concentrations of coprostanol and fecal indicator bacteria in tropical (Mekong delta) and temperate (Tokyo) freshwaters. *Appl. Environ. Microbiol.* 70 814–821 10.1128/AEM.70.2.814-821.2004 14766559 PMC348936

[B53] Isobe K. O. Tarao M. Zakaria M. P. Chiem N. H. Minh L. Y. Takada H. (2002). Quantitative application of fecal sterols using gas chromatography-mass spectrometry to investigate fecal pollution in tropical waters: Western Malaysia and Mekong Delta, Vietnam. *Environ. Sci. Technol.* 36 4497–4507 10.1021/es020556h 12433157

[B54] Jaffrezic A. Jarde E. Pourcher A. M. Gourmelon M. Caprais M. P. Heddadj D. (2011). Microbial and chemical markers: runoff transfer in animal manure-amended soils. *J. Environ. Qual.* 40 959–968 10.2134/jeq2010.0355 21546682

[B55] Jagai J. S. Castronovo D. A. Monchak J. Naumova E. N. (2009). Seasonality of cryptosporidiosis: a meta-analysis approach. *Environ. Res.* 109 465–478 10.1016/j.envres.2009.02.008 19328462 PMC2732192

[B56] Janeau J. L. Gillard L. C. Grellier S. Jouquet P. Le T. P. Q. Luu T. N. M. (2014). Soil erosion, dissolved organic carbon and nutrient losses under different land use systems in a small catchment in northern Vietnam. *Agric. Water Manage.* 146 314–323 10.1016/j.agwat.2014.09.006

[B57] Jarde E. Gruau G. Mansuy-Huault L. (2005). The coprostanol/sterol ratio as indicator of organic matter provenance in soils and rivers. *Geochim. Cosmochim. Acta* 69 A756–A756.

[B58] Jarde E. Gruau G. Mansuy-Huault L. (2007). Detection of manure-derived organic compounds in rivers draining agricultural areas of intensive manure spreading. *Appl. Geochem.* 22 1814–1824 10.1016/j.apgeochem.2007.03.037

[B59] Jeanneau L. Jarde E. Gruau G. (2011). Influence of salinity and natural organic matter on the solid phase extraction of sterols and stanols: application to the determination of the human sterol fingerprint in aqueous matrices. *J. Chromatogr. A* 1218 2513–2520 10.1016/j.chroma.2011.02.066 21420686

[B60] Jeanneau L. Solecki O. Wery N. Jardé E. Gourmelon M. Communal P. Y. (2012). Relative decay of fecal indicator bacteria and human-associated markers: a microcosm study simulating wastewater input into seawater and freshwater. *Environ. Sci. Technol.* 46 2375–2382 10.1021/es203019y 22236067

[B61] Jiminez L. Muniz I. Toranzos G. A. Hazen T. C. (1989). Survival and activity of *Salmonella typhimurium* and *Escherichia coli* in tropical freshwater. *J. Appl. Bacteriol.* 67 61–69 10.1111/j.1365-2672.1989.tb04955.x 2674097

[B62] Kaisermann A. Roguet A. Nunan N. Maron P.-A. Ostle N. Lata J.-C. (2013). Agricultural management affects the response of soil bacterial community structure and respiration to water-stress. *Soil Biol. Biochem.* 66 69–77 10.1016/j.soilbio.2013.07.001

[B63] Karanis P. Kourenti C. Smith H. (2007). Waterborne transmission of protozoan parasites: a worldwide review of outbreaks and lessons learnt. *J. Water Health* 5 1–38 10.2166/wh.2006.002 17402277

[B64] Karim M. R. Manshadi F. D. Karpiscak M. M. Gerba C. P. (2004). The persistence and removal of enteric pathogens in constructed wetlands. *Water Res.* 38 1831–1837 10.1016/j.watres.2003.12.029 15026238

[B65] Kashefipour S. M. Lin B. Harris E. Falconer R. A. (2002). Hydro-environmental modelling for bathing water compliance of an estuarine basin. *Water Res.* 36 1854–1868 10.1016/S0043-1354(01)00396-7 12044085

[B66] Kim J. W. Pachepsky Y. A. Shelton D. R. Coppock C. (2010). Effect of streambed bacteria release on *E. coli* concentrations: monitoring and modeling with the modified SWAT. *Ecol. Modell.* 221 1592–1604 10.1016/j.ecolmodel.2010.03.005

[B67] Kimani-Murage E. Ngindu A. (2007). Quality of water the slum dwellers use: the case of a Kenyan slum. *J. Urban Health* 84 829–838 10.1007/s11524-007-9199-x 17551841 PMC2134844

[B68] Klaus J. McDonnell J. (2013). Hydrograph separation using stable isotopes: review and evaluation. *J. Hydrol.* 505 47–64 10.1016/j.jhydrol.2013.09.006

[B69] Kloot R. W. (2007). Locating *Escherichia coli* contamination in a rural South Carolina watershed. *J. Environ. Manage.* 83 402–408 10.1016/j.jenvman.2006.03.008 16814453

[B70] Korajkic A. Mcminn B. R. Harwood V. J. Shanks O. C. Fout G. S. Ashbolt N. J. (2013a). Differential decay of enterococci and *Escherichia coli* originating from two fecal pollution sources. *Appl. Environ. Microbiol.* 79 2488–2492 10.1128/AEM.03781-12 23377944 PMC3623250

[B71] Korajkic A. Wanjugi P. Harwood V. J. (2013b). Indigenous microbiota and habitat influence *Escherichia coli* survival more than sunlight in simulated aquatic environments. *Appl. Environ. Microbiol.* 79 5329–5337 10.1128/AEM.01362-13 23811514 PMC3753954

[B72] Korajkic A. Mcminn B. R. Shanks O. C. Sivaganesan M. Fout G. S. Ashbolt N. J. (2014). Biotic interactions and sunlight affect persistence of fecal indicator bacteria and microbial source tracking genetic markers in the upper Mississippi river. *Appl. Environ. Microbiol.* 80 3952–3961 10.1128/AEM.00388-14 24747902 PMC4054226

[B73] Ksoll W. B. Ishii S. Sadowsky M. J. Hicks R. E. (2007). Presence and sources of fecal coliform bacteria in epilithic periphyton communities of lake superior. *Appl. Environ. Microbiol.* 73 3771–3778 10.1128/AEM.02654-06 17468280 PMC1932738

[B74] Kunii O. Nakamura S. Abdur R. Wakai S. (2002). The impact on health and risk factors of the diarrhoea epidemics in the 1998 Bangladesh floods. *Public Health* 116 68–74 10.1016/S0033-3506(02)00506-1 11961673

[B75] Lal R. (1983). “Soil erosion in the humid tropics with particular reference to agricultural land development and soil management,” in *Proceeding of Symposium on the Hydrology of Humid Tropical Regions with Particular Reference to the Hydrological Effects of Agriculture and Forestry Practice* ed. Keller R. (Hamburg: IAHS) 221–240.

[B76] Le T. P. Q. Billen G. Garnier J. Thery S. Fezard C. Minh C. V. (2005). Nutrient (N, P) budgets for the Red River basin (Vietnam and China). *Global Biogeochem. Cycles* 19 GB2022.

[B77] Lee K. Y. Fisher T. R. Rochelle-Newall E. (2001). Modeling the hydrochemistry of the Choptank river basin using GWLF and Arc/Info: 2. Model validation and application. *Biogeochemistry* 56 311–348 10.1023/A:1013169027082

[B78] Lemee R. Rochelle-Newall E. Van Wambeke F. Pizay M. D. Rinaldi P. Gattuso J. P. (2002). Seasonal variation of bacterial production, respiration and growth efficiency in the open NW Mediterranean Sea. *Aquat. Microb. Ecol.* 29 227–237 10.3354/ame029227

[B79] Liang Z. He Z. Zhou X. Powell C. A. Yang Y. He L. M. (2013). Impact of mixed land-use practices on the microbial water quality in a subtropical coastal watershed. *Sci. Total Environ.* 449 426–433 10.1016/j.scitotenv.2013.01.087 23454704

[B80] Luu T. N. M. Garnier J. Billen G. Orange D. Nemery J. Le T. P. Q. (2010). Hydrological regime and water budget of the Red River Delta (Northern Vietnam). *J. Asian Earth Sci.* 37 219–228 10.1016/j.jseaes.2009.08.004

[B81] Magdoff F. Weil R. R. (2004). “Soil organic matter management strategies,” in *Soil Organic Matter in Sustainable Agriculture* eds Magdoff F. Weil R. R. (London: CRC Press) 45–66 10.1201/9780203496374

[B82] Mahloch J. (1974). Comparative analysis of modeling techniques for coliform organisms in streams. *Appl. Microbiol.* 27 340–345. 4595960 10.1128/am.27.2.340-345.1974 PMC380032

[B83] McDaniel R. L. Soupir M. L. Tuttle R. B. Cervantes A. E. (2013). Release, dispersion, and resuspension of *Escherichia coli* from direct fecal deposits under controlled flows. *J. Am. Water Resour. Assoc.* 49 319–327 10.1111/jawr.12022

[B84] Menon P. Billen G. Servais P. (2003). Mortality rates of autochthonous and fecal bacteria in natural aquatic ecosystems. *Water Res.* 37 4151–4158 10.1016/S0043-1354(03)00349-X 12946897

[B85] Mieszkin S. Furet J. Corthier G. Gourmelon M. (2009). Estimation of pig fecal contamination in a river catchment by real-time PCR using two pig-specific Bacteroidales 16S rRNA genetic markers. *Appl. Environ. Microbiol.* 75 3045–3054 10.1128/AEM.02343-08 19329663 PMC2681621

[B86] Miller C. T. Dawson C. N. Farthing M. W. Hou T. Y. Huang J. Kees C. E. (2013). Numerical simulation of water resources problems: models, methods, and trends. *Adv. Water Resour.* 51 405–437 10.1016/j.advwatres.2012.05.008

[B87] Milliman J. D. (1995). Sediment discharge to the ocean from small mountainous rivers: the New Guinea example. *Geo Mar. Lett.* 15 127–133 10.1007/BF01204453

[B88] Milliman J. D. Meade R. H. (1983). World-wide delivery of river sediment to the oceans. *J. Geol.* 91 1–21 10.1086/628741

[B89] Milliman J. D. Syvitski J. P. M. (1992). Geomorphic tectonic control of sediment discharge into the ocean: the importance of small mountainous rivers. *J. Geol.* 100 525–544 10.1086/629606

[B90] Mitch A. A. Gasner K. C. Mitch W. A. (2010). Fecal coliform accumulation within a river subject to seasonally-disinfected wastewater discharges. *Water Res.* 44 4776–4782 10.1016/j.watres.2010.05.060 20580053

[B91] Monaghan R. M. Carey P. L. Wilcock R. J. Drewry J. J. Houlbrooke D. J. Quinn J. M. (2009). Linkages between land management activities and stream water quality in a border dyke-irrigated pastoral catchment. *Agric. Ecosyst. Environ.* 129 201–211 10.1016/j.agee.2008.08.017

[B92] Navratil O. Legout C. Gateuille D. Esteves M. Liebault F. (2010). Assessment of intermediate fine sediment storage in a braided river reach (southern French Prealps). *Hydrol. Process.* 24 1318–1332 10.1002/hyp.7594

[B93] Neave M. Luter H. Padovan A. Townsend S. Schobben X. Gibb K. (2014). Multiple approaches to microbial source tracking in tropical northern Australia. *Microbiologyopen* 3 860–874 10.1002/mbo3.209 25224738 PMC4263510

[B94] Nichols G. Lane C. Asgari N. Verlander N. Q. Charlett A. (2009). Rainfall and outbreaks of drinking water related disease and in England and Wales. *J. Water Health* 7 1–8 10.2166/wh.2009.143 18957770

[B95] Nshimyimana J. P. Ekklesia E. Shanahan P. Chua L. H. C. Thompson J. R. (2014). Distribution and abundance of human-specific Bacteroides and relation to traditional indicators in an urban tropical catchment. *J. Appl. Microbiol.* 116 1369–1383 10.1111/jam.12455 24460587 PMC4271309

[B96] Oliver D. M. Clegg C. D. Heathwaite A. L. Haygarth P. M. (2007). Preferential attachment of *Escherichia coli* to different particle size fractions of an agricultural grassland soil. *Water Air Soil Pollut.* 185 369–375 10.1007/s11270-007-9451-8

[B97] Oliver D. M. Haygarth P. M. Clegg C. D. Heathwaite L. (2006). Differential *E. coli* die-off patterns associated with agricultural matrices. *Environ. Sci. Technol.* 40 5710–5716 10.1021/es0603249 17007130

[B98] Opisa S. Odiere M. R. Jura W. G. Z. O. Diana M. S. Karanja D. M. S. Pauline N. M. (2012). Faecal contamination of public water sources in informal settlements of Kisumu City, western Kenya. *Water Sci. Technol.* 66 2674–2681 10.2166/wst.2012.503 23109585

[B99] Otto K. Elwing H. Hermansson M. (1999). Effect of ionic strength on initial interactions of *Escherichia coli* with surfaces, studied on-line by a novel quartz crystal microbalance technique. *J. Bacteriol.* 181 5210–5218. 10464189 10.1128/jb.181.17.5210-5218.1999 PMC94024

[B100] Pachepsky Y. A. Sadeghi A. M. Bradford S. A. Shelton D. R. Guber A. K. Dao T. (2006). Transport and fate of manure-borne pathogens: modeling perspective. *Agric. Water Manage.* 86 81–92 10.1016/j.agwat.2006.06.010

[B101] Pachepsky Y. A. Shelton D. R. (2011). Escherichia coli and Fecal Coliforms in freshwater and estuarine Sediments. *Crit. Rev. Environ. Sci. Technol.* 41 1067–1110 10.1080/10643380903392718

[B102] Pachepsky Y. Shelton D. R. Mclain J. E. T. Patel J. Mandrell R. E. (2011). “Irrigation waters as a source of pathogenic microorganisms in produce: a review,” in *Advances in Agronomy* ed. Sparks D. L. (Burlington, VT: Academic Press) 73–141 10.1016/B978-0-12-386473-4.00002-6

[B103] Pandey P. K. Soupir M. L. (2013). Assessing the impacts of *E.coli* laden streambed sediment on E.coli loads over a range of flows and sediment characteristics. *J. Am. Water Resour. Assoc.* 49 1261–1269 10.1111/jawr.12079

[B104] Pandey P. K. Soupir M. L. Haddad M. Rothwell J. J. (2012a). Assessing the impacts of watershed indexes and precipitation on spatial in-stream *E. coli* concentrations. *Ecol. Indic.* 23 641–652 10.1016/j.ecolind.2012.05.023

[B105] Pandey P. K. Soupir M. L. Rehmann C. R. (2012b). A model for predicting resuspension of *Escherichia coil* from streambed sediments. *Water Res.* 46 115–126 10.1016/j.watres.2011.10.019 22082528

[B106] Pandey P. K. Soupir M. L. Ikenberry C. (2014). “Modelling animal waste pathogen transport from agricultural land to streams,” in *Proceedings of the International Conferences on Geological, Geographical, Aerospace and Earth Sciences* Jakarta.

[B107] Patin J. Mouche E. Ribolzi O. Chaplot V. Sengtahevanghoung O. Latsachak K. O. (2012). Analysis of runoff production at the plot scale during a long-term survey of a small agricultural catchment in Lao PDR. *J. Hydrol.* 426 79–92 10.1016/j.jhydrol.2012.01.015

[B108] Planchon O. Cadet P. Lapetite J. M. Silvera N. Esteves M. (2000). Relationship between raindrop erosion and runoff erosion under simulated rainfall in the Sudano-Sahel: consequences for the spread of nematodes by runoff. *Earth Surf. Process Land.* 25 729–741 10.1002/1096-9837(200007)25:7<729::AID-ESP128>3.0.CO;2-C

[B109] Podwojewski P. Orange D. Jouquet P. Valentin C. Nguyen V. T. Janeau J. L. (2008). Land-use impacts on surface runoff and soil detachment within agricultural sloping lands in Northern Vietnam. *Catena* 74 109–118 10.1016/j.catena.2008.03.013

[B110] Pommier T. Canback B. Riemann L. Bostrom K. H. Simu K. Lundberg P. (2007). Global patterns of diversity and community structure in marine bacterioplankton. *Mol. Ecol.* 16 867–880 10.1111/j.1365-294X.2006.03189.x 17284217

[B111] Pommier T. Merroune A. Bettarel Y. Got P. Janeau J.-L. Jouquet P. (2014). Off-site impacts of agricultural composting: role of terrestrially derived organic matter in structuring aquatic microbial communities and their metabolic potential. *FEMS Microbiol. Ecol.* 90 622–632 10.1111/1574-6941.12421 25195703

[B112] Ratajczak M. Laroche E. Berthe T. Clermont O. Pawlak B. Denamur E. (2010). Influence of hydrological conditions on the *Escherichia coli* population structure in the water of a creek on a rural watershed. *BMC Microbiol.* 10:222 10.1186/1471-2180-10-222 PMC2933670 20723241

[B113] Regina V. R. Lokanathan A. R. Modrzyński J. J. Sutherland D. S. Meyer R. L. (2014). Surface phsicochemistry and ionic strength affects eDNA’s role in bacterial adhesion to abiotic surfaces. *PLoS ONE* 9:e105033 10.1371/journal.pone.0105033 PMC4133339 25122477

[B114] Ribolzi O. Cuny J. Sengsoulichanh P. Mousques C. Soulileuth B. Pierret A. (2011a). Land use and water quality along a mekong tributary in Northern Lao PDR. *Environ. Manage.* 47 291–302 10.1007/s00267-010-9593-0 21132547

[B115] Ribolzi O. Patin J. Bresson L. M. Latsachack K. O. Mouche E. Sengtaheuanghoung O. (2011b). Impact of slope gradient on soil surface features and infiltration on steep slopes in northern Laos. *Geomorphology* 127 53–63 10.1016/j.geomorph.2010.12.004

[B116] Rivera S. C. Hazen T. C. Toranzos G. A. (1988). Isolation of fecal coliforms from pristine sites in a tropical rain forest. *Appl. Environ. Microbiol.* 54 513–517. 3281583 10.1128/aem.54.2.513-517.1988 PMC202482

[B117] Rochelle-Newall E. Hulot F. D. Janeau J. L. Merroune A. (2014). CDOM fluorescence as a proxy of DOC concentration in natural waters: a comparison of four contrasting tropical systems. *Environ. Monit. Assess.* 186 589–596 10.1007/s10661-013-3401-2 24072524

[B118] September S. M. Els F. A. Venter S. N. Brözel V. S. (2007). Prevalence of bacterial pathogens in biofilms of drinking water distribution systems. *J. Water Health* 5 219–227. 17674571

[B119] Sinclair A. Hebb D. Jamieson R. Gordon R. Benedict K. Fuller K. (2009). Growing season surface water loading of fecal indicator organisms within a rural watershed. *Water Res.* 43 1199–1206 10.1016/j.watres.2008.12.006 19117588

[B120] Sinton L. W. Hall C. H. Lynch P. A. Davies-Colley R. J. (2002). Sunlight inactivation of fecal indicator bacteria and bacteriophages from waste stabilization pond eﬄuent in fresh and saline waters. *Appl. Environ. Microbiol.* 68 1122–1131 10.1128/AEM.68.3.1122-1131.2002 11872459 PMC123754

[B121] Smith C. D. Berk S. G. Brandl M. T. Riley L. W. (2012). Survival characteristics of diarrheagenic *Escherichia coli* pathotypes and *Helicobacter* pylori during passage through the free-living ciliate, *Tetrahymena* sp. *FEMS Microbiol. Ecol.* 82 574–583 10.1111/j.1574-6941.2012.01428.x 22680607

[B122] Solecki O. Jeanneau L. Jarde E. Gourmelon M. Marin C. Pourcher A. M. (2011). Persistence of microbial and chemical pig manure markers as compared to faecal indicator bacteria survival in freshwater and seawater microcosms. *Water Res.* 45 4623–4633 10.1016/j.watres.2011.06.012 21745675

[B123] Solo-Gabriele H. M. Wolfert M. A. Desmarais T. R. Palmer C. J. (2000). Sources of *Escherichia coli* in a coastal subtropical environment. *Appl. Environ. Microbiol.* 66 230–237 10.1128/AEM.66.1.230-237.2000 10618229 PMC91811

[B124] Soupir M. L. Mostaghimi S. (2011). *Escherichia coli* and Enterococci attachment to particles in runoff from highly and sparsely vegetated grassland. *Water Air Soil Pollut.* 216 167–178 10.1007/s11270-010-0524-8

[B125] Staley Z. R. Rohr J. R. Harwood V. J. (2011). Test of direct and indirect effects of agrochemicals on the survival of fecal indicator bacteria. *Appl. Environ. Microbiol.* 77 8765–8774 10.1128/AEM.06044-11 22003017 PMC3233103

[B126] Staley Z. R. Rohr J. R. Senkbeil J. K. Harwood V. J. (2014). Agrochemicals indirectly increase survival of *E. coli* O157:H7 and indicator bacteria by reducing ecosystem services. *Ecol. Appl.* 24 1945–1953 10.1890/13-1242.1 29185664

[B127] Staley Z. R. Senkbeil J. K. Rohr J. R. Harwood V. J. (2012). Lack of direct effects of agrochemicals on zoonotic pathogens and fecal indicator bacteria. *Appl. Environ. Microbiol.* 78 8146–8150 10.1128/AEM.01815-12 22961900 PMC3485959

[B128] Strauch A. M. Mackenzie R. A. Bruland G. L. Tingley R. Giardina C. P. (2014). Climate change and land use drivers of fecal bacteria in tropical Hawaiian rivers. *J. Environ. Qual.* 43 1475–1483 10.2134/jeq2014.01.0025 25603095

[B129] Stumpf C. H. Piehler M. F. Thompson S. Noble R. T. (2010). Loading of fecal indicator bacteria in North Carolina tidal creek headwaters: hydrographic patterns and terrestrial runoff relationships. *Water Res.* 44 4704–4715 10.1016/j.watres.2010.07.004 20673947

[B130] Suter E. Juhl A. O’mullan G. (2011). Particle association of *Enterococcus* and total Bacteria in the Lower Hudson River Estuary, USA. *J. Water Resour. Prot.* 3 715–725 10.4236/jwarp.2011.310082

[B131] Thoe W. Wong S. H. C. Choi K. W. Lee J. H. W. (2012). Daily prediction of marine beach water quality in Hong Kong. *J. Hydro Environ. Res.* 6 164–180 10.1016/j.jher.2012.05.003

[B132] Thothong W. Huon S. Janeau J. L. Boonsaner A. De Rouw A. Planchon O. (2011). Impact of land use change and rainfall on sediment and carbon accumulation in a water reservoir of North Thailand. *Agric. Ecosyst. Environ.* 140 521–533 10.1016/j.agee.2011.02.006

[B133] Topp E. Welsh M. Tien Y. C. Dang A. Lazarovits G. Conn K. (2003). Strain-dependent variability in growth and survival of *Escherichia coli* in agricultural soil. *FEMS Microbiol. Ecol.* 44 303–308 10.1016/S0168-6496(03)00055-2 19719611

[B134] Trevisan D. Vansteelant J. Y. Dorioz J. M. (2002). Survival and leaching of fecal bacteria after slurry spreading on mountain hay meadows: consequences for the management of water contamination risk. *Water Res.* 36 275–283 10.1016/S0043-1354(01)00184-1 11766805

[B135] Troussellier M. Got P. Bouvy M. M’Boup M. Arfi R. Lebihan F. (2004). Water quality and health status of the Senegal River estuary. *Mar. Pollut. Bull.* 48 852–862 10.1016/j.marpolbul.2003.10.028 15111032

[B136] Vidon P. Campbell M. A. Gray M. (2008a). Unrestricted cattle access to streams and water quality in till landscape of the Midwest. *Agric. Water Manage.* 95 322–330 10.1016/j.agwat.2007.10.017

[B137] Vidon P. Tedesco L. P. Wilson J. Campbell M. A. Casey L. R. Gray M. (2008b). Direct and indirect hydrological controls on *E. coli* concentration and loading in Midwestern streams. *J. Environ. Qual.* 37 1761–1768 10.2134/jeq2007.0311 18689737

[B138] Vigiak O. Ribolzi O. Pierret A. Sengtaheuanghoung O. Valentin C. (2008). Trapping efficiencies of cultivated and natural riparian vegetation of northern Laos. *J. Environ. Qual.* 37 889–897 10.2134/jeq2007.0251 18453411

[B139] Vijayavel K. Sadowsky M. J. Ferguson J. A. Kashian D. R. (2013). The establishment of the nuisance cyanobacteria *Lyngbya* wollei in Lake St. Clair and its potential to harbor fecal indicator bacteria. *J. Great Lakes Res.* 39 560–568 10.1016/j.jglr.2013.09.018

[B140] Wanjugi P. Harwood V. J. (2013). The influence of predation and competition on the survival of commensal and pathogenic fecal bacteria in aquatic habitats. *Environ. Microbiol.* 15 517–526 10.1111/j.1462-2920.2012.02877.x 23013262

[B141] Wanyama J. Herremans K. Maetens W. Isabirye M. Kahimba F. Kimaro D. (2012). Effectiveness of tropical grass species as sediment filters in the riparian zone of Lake Victoria. *Soil Use Manage.* 28 409–418 10.1111/j.1475-2743.2012.00409.x

[B142] WHO. (2012). *World Health Organisation Global Data Repository*. Available at: apps.who.int/ghodata/: World Health Organisation

[B143] Wilkinson J. Jenkins A. Wyer M. Kay D. (1995). Modelling faecal coliform dynamics in streams and rivers. *Water Res.* 29 847–855 10.1016/0043-1354(94)00211-O

[B144] Winfield M. D. Groisman E. A. (2003). Role of nonhost environments in the lifestyles of *Salmonella* and *Escherichia coli*. *Appl. Environ. Microbiol.* 69 3687–3694 10.1128/AEM.69.7.3687-3694.2003 12839733 PMC165204

[B145] Wingender J. Flemming H.-C. (2011). Biofilms in drinking water and their role as reservoir for pathogens. *Int. J. Hyg. Environ. Health* 214 417–423 10.1016/j.ijheh.2011.05.009 21697011

[B146] Yajima A. Kurokura H. (2008). Microbial risk assessment of livestock-integrated aquaculture and fish handling in Vietnam. *Fish. Sci.* 74 1062–1068 10.1111/j.1444-2906.2008.01625.x

[B147] Zhang W. W. Li H. Sun D. F. Zhou L. D. (2012). A statistical assessment of the impact of agricultural land use intensity on regional surface water quality at multiple scales. *Int. J. Environ. Res. Public Health* 9 4170–4186 10.3390/ijerph9114170 23202839 PMC3524620

[B148] Ziegler A. D. Negishi J. Sidle R. C. Preechapanya P. Sutherland R. A. Giambelluca T. W. (2006). Reduction of stream sediment concentration by a riparian buffer: filtering of road runoff in disturbed headwater basins of montane mainland southeast Asia. *J. Environ. Qual.* 35 151–162 10.2134/jeq2005.0103 16391286

[B149] Zita A. Hermansson M. (1994). Effects of ionic strength on bacterial adhesion and stability of flocs in a wastewater activated sludge system. *Appl. Environ. Microbiol.* 60 3041–3048. 16349365 10.1128/aem.60.9.3041-3048.1994 PMC201769

[B150] Zoetendal E. G. Raes J. Van Den Bogert B. Arumugam M. Booijink C. C. G. M. Troost F. J. (2012). The human small intestinal microbiota is driven by rapid uptake and conversion of simple carbohydrates. *ISME J.* 6 1415–1426 10.1038/ismej.2011.212 22258098 PMC3379644

